# pycofitness—Evaluating the fitness landscape of RNA and protein sequences

**DOI:** 10.1093/bioinformatics/btae074

**Published:** 2024-02-09

**Authors:** Fabrizio Pucci, Mehari B Zerihun, Marianne Rooman, Alexander Schug

**Affiliations:** Computational Biology and Bioinformatics, Université Libre de Bruxelles, 1050 Brussels, Belgium; Interuniversity Institute of Bioinformatics in Brussels, 1050 Brussels, Belgium; John von Neumann Institute for Computing, Jülich Supercomputer Centre, 52428 Jülich, Germany; Computational Biology and Bioinformatics, Université Libre de Bruxelles, 1050 Brussels, Belgium; Interuniversity Institute of Bioinformatics in Brussels, 1050 Brussels, Belgium; John von Neumann Institute for Computing, Jülich Supercomputer Centre, 52428 Jülich, Germany; Department of Biology, University of Duisburg-Essen, D-45141 Essen, Germany

## Abstract

**Motivation:**

The accurate prediction of how mutations change biophysical properties of proteins or RNA is a major goal in computational biology with tremendous impacts on protein design and genetic variant interpretation. Evolutionary approaches such as coevolution can help solving this issue.

**Results:**

We present pycofitness, a standalone Python-based software package for the *in silico* mutagenesis of protein and RNA sequences. It is based on coevolution and, more specifically, on a popular inverse statistical approach, namely direct coupling analysis by pseudo-likelihood maximization. Its efficient implementation and user-friendly command line interface make it an easy-to-use tool even for researchers with no bioinformatics background. To illustrate its strengths, we present three applications in which pycofitness efficiently predicts the deleteriousness of genetic variants and the effect of mutations on protein fitness and thermodynamic stability.

**Availability and implementation:**

https://github.com/KIT-MBS/pycofitness.

## 1 Introduction

Accurately predicting the impact of mutations on protein and RNA stability and function is a longstanding issue in computational biology. Achieving this goal would be beneficial for a huge series of applications such as protein design (Coluzza 2017, Pucci *et al.* 2022), interpretation of genetic variants (Gerasimavicius *et al.* 2020, Iqbal *et al.* 2020) and understanding antibiotic resistance (Woodford and Ellington 2007). The massive amount of sequences made available over the past decade through the advancement of next-generation sequencing technologies can be exploited to estimate the effect of mutations. Indeed, conserved sites in multiple sequence alignments (MSA) of homologous proteins or RNAs typically characterize functionally or structurally important sites. Therefore, mutations at these sites are not well tolerated and generally removed during evolution.

Coevolutionary approaches, in which correlations between pairs of sites in MSAs are considered, are an additional level of evolutionary information that can be extracted from sequence data. Direct coupling analysis (DCA) (Schug *et al.* 2009, Weigt *et al.* 2009, Morcos *et al.* 2011) is one of these methods. It uses an inverse statistical inference formalism to extract coevolutionary information and to identify pairs of MSA sites that coevolve.

Coevolutionary approaches and the information extracted from them have been extensively used as constraints in 3D structure modeling of proteins (Schug *et al.* 2009, Marks *et al.* 2011, Dago *et al.* 2012, Hopf *et al.* 2012, Morcos *et al.* 2013) and RNA (De Leonardis *et al.* 2015, Weinreb *et al.* 2016, Pucci and Schug 2019). They have also been employed in the computational characterization of the impact of mutations on protein fitness and biophysical characteristics and provide an efficient estimation of deep mutagenesis scanning data (Cheng *et al.* 2016, Figliuzzi *et al.* 2016, Flynn *et al.* 2017, Hopf *et al.* 2017, Peng *et al.* 2019, Frazer *et al.* 2021). Recently, they have been successfully used in the construction of generative protein sequence models for protein design applications (Russ *et al.* 2020, McGee *et al.* 2021, Trinquier *et al.* 2021).

To summarize, coevolutionary methods are of fundamental interest in a broad array of applications, including the understanding of genetic variant deleteriousness and, from a biotechnological perspective, the rational design of proteins and RNA.

## 2 Direct coupling analysis

DCA is a statistical method to extract coevolutionary information from an MSA of homologous RNA or protein sequences (Cocco *et al.* 2018). Let P(S) be the probability that a given RNA (protein) sequence $S=a_{1}a_{2}\ldots a_{L}$ of length *L*, in which each state $a_{i}$ is either an RNA base (an amino acid residue) or a gap, is sampled over the course of evolution. P(S) can be written using the Boltzmann law as:

(1) $$P(S)=\frac{1}{Z}\text{exp}(-\beta \phi ),$$

where β is the inverse of the temperature, *Z* the partition function of the model and ϕ the Hamiltonian of the system, which in turn is expressed via a generalized Potts model as:

(2) $$\phi =-\sum_{i<j}^{L}J_{ij}(a_{i},a_{j})-\sum_{i=1}^{L}h_{i}(a_{i}).$$

The parameters $h_{i}(a_{i})$ measure the local field strength at site *i* occupied by state $a_{i}$ and the coupling parameters $J_{ij}(a_{i},a_{j})$ quantify the coupling strength between pairs of sites *i* and *j* occupied by states $a_{i}$ and $a_{j}$, respectively.

The local field and the coupling parameters of the model are inferred from the MSA of RNAs (proteins) that are homologous to *S* using inverse statistical algorithms such as message passing DCA (mpDCA) (Weigt *et al.* 2009), mean-field DCA (mfDCA) (Morcos *et al.* 2011), pseudo-likelihood maximization DCA (plmDCA) (Ekeberg *et al.* 2013), Boltzmann learning (Cuturello *et al.* 2020), Gaussian DCA (Baldassi *et al.* 2014) and autoregressive DCA (arDCA) (Trinquier *et al.* 2021).

Let us now consider a substitution at position *i* in the sequence *S*, with state $a_{i}$ replaced by $b_{i}$. We can quantify the effect of the substitution on the evolutionary energy, $\Delta E(a_{i}\to b_{i})$, by computing the change in ϕ between the wild type and mutated RNA (protein) as:

(3) $$\Delta E(a_{i}\to b_{i})=\phi (a_{1}\ldots a_{i}\ldots a_{L})-\phi (a_{1}\ldots b_{i}\ldots a_{L}).$$

With this convention, the lower the ΔE, the more deleterious the mutation is for the RNA (protein). Conversely, if ΔE is positive, the mutation is beneficial for the protein. In terms of the coupling and single-site field strengths, Equation (3) can be rewritten as:

(4) $$\Delta E(a_{i}\to b_{i})=h_{i}(b_{i})-h_{i}(a_{i})+\sum_{j=1,j\neq i}^{L}\left(J_{ij}(b_{i},a_{j})-J_{ij}(a_{i},a_{j})\right).$$

This change in evolutionary energy is a measure of the fitness of the mutated RNA (protein) with respect to the wild type.

## 3 Implementation of pycofitness

We have developed pycofitness, a Python package to perform *in silico* mutagenesis experiments of protein or RNA sequences using a widely recognized DCA approach. Based on an input MSA of proteins or RNAs homologous to the target sequence, pycofitnesss calculates the couplings and single-site fields of a coevolutionary model employing the plmDCA algorithm that we have implemented earlier in the python package pyDCA (Zerihun *et al.* 2020). The strengths of plmDCA with respect to other DCA approaches are demonstrated in Supplementary Section S3. Details about the implementation and use of pycofitness are given in Supplementary Section S1; for technical details about the model and parameter choice, such as the regularization terms, we refer the reader to Zerihun *et al.* (2020).

Once the model is inferred, pycofitness inserts, in turn, all possible single-site mutations in the reference sequence, i.e. three nucleotide substitutions per site for RNAs and 19 amino acid substitutions for proteins. pycofitness estimates the effect of a nucleotide or amino acid substitution as in Equation (4), thus by computing the difference in evolutionary energy between the wild-type and mutated sequences, using the inferred coevolutionary model.

The pycofitness software has several strengths and capabilities:

- First and foremost, it is a standalone Python software that can be easily installed from the Python packaging index (PyPI). It can be used through the command line or as a Python library. Its user interface can be effortlessly imported and integrated with user-developed Python source codes. Details are given in Supplementary Section S2.
- Moreover, the computationally demanding parts of the algorithm, such as parameter inference through plmDCA, are implemented using the C/C++ backend, enabling efficient code parallelization and allowing pycofitness to perform long protein and RNA mutagenesis in a reasonable amount of time.
- Another useful feature of pycofitness is its ability to perform RNA mutagenesis, a field of growing interest. Only a few tools are available for such studies, making pycofitness an invaluable addition to the current research landscape.
- pycofitness is also user-friendly: the only input it requires is the MSA and the type of biomolecule (“RNA” or “protein”).

## 4 Applications of pycofitness

In addition to its ease of use, pycofitness has been extensively tested for its accuracy, as shown below and in Supplementary Section S2. Moreover, a novelty and advantage of pycofitness is that it can run coevolutionary mutagenesis analyses of both protein and RNA sequences, unlike similar packages such as EVcoupling (Hopf *et al.* 2018).

Here we applied pycofitness to non-coding RNA molecules, and in particular, tRNAs, which are essential molecules in the protein synthesis pathway and link the genetic information to the amino acid sequence of proteins. More specifically, we studied the effect of mutations on mitochondrially encoded tRNAs: tRNA-Phe (MT-TF), tRNA-Val (MT-TV), tRNA-Leu I (MT-TL1), and tRNA-Ile (MT-TI).

We started by collecting variants in these four genes from the ClinVar database (Landrum *et al.* 2018). We considered two classes of variants: the pathogenic class containing the variants assigned as pathogenic and likely pathogenic, and the benign class grouping likely benign and benign variants. Variants in these genes have been related to a wide variety of genetic diseases such as mitochondrial encephalomyopathy, lactic acidosis, stroke-like episodes (MELAS), myoclonic epilepsy with ragged-red fibers (MERRF), mitochondrial cardiomyopathy, and complex IV deficiency of the mitochondrial respiratory chain (Abbott *et al.* 2014).

We computed the change in coevolutionary energy ΔE of the variants and used it to predict their deleteriousness. We started by aligning the tRNA sequences from the human reference genome GRCh38 with the RFAM family RF00005 (Kalvari *et al.* 2021) using the RNA homologous sequence inference model Infernal (Nawrocki and Eddy 2013). Once the MSA was constructed, we run pycofitness to compute the ΔE values. If ΔE is below a given threshold value, which was optimized for each gene in leave-one-out cross validation, the variants were considered as pathogenic; otherwise the variants were predicted as benign.

We used common metrics for binary classification to assess the accuracy of pycofitness, i.e. sensitivity [TP/(TP+FN)] and specificity [TN/(TN+FP)], where TP are the variants correctly predicted as pathogenic, TN, those correctly predicted as benign, FP, those wrongly classified as pathogenic and FN, those wrongly predicted as benign. We also computed the balanced accuracy (BACC) as the average between sensitivity and specificity, as well as the threshold-independent metric that is the area under the receiver operating characteristic curve (AUC-ROC).

**Figure 1.**
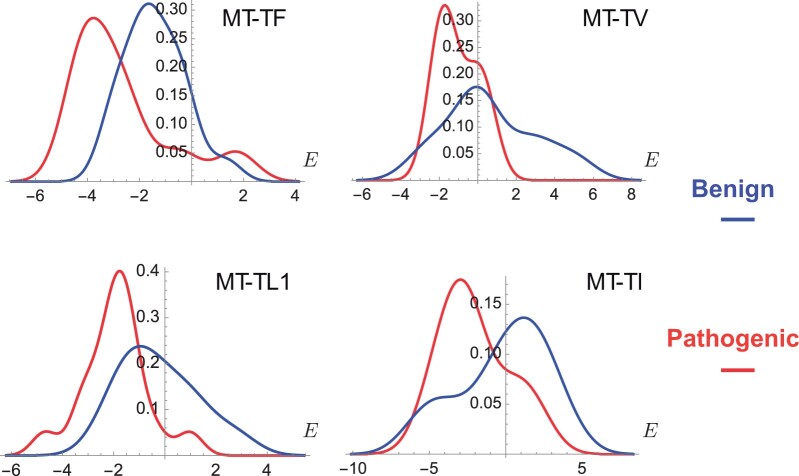
Probability distribution of the change in coevolutionary energy ΔE for pathogenic and benign variants in four mitochondrially encoded tRNAs: tRNA-Phe (MT-TF), tRNA-Val (MT-TV), tRNA-Leu I (MT-TL1), and tRNA-Ile (MT-TI).

As shown in Table 1, pycofitness is able to predict the deleteriousness of the variants in the tested non-coding RNAs with good accuracy, with BACC scores between 0.63 and 0.79 and AUC-ROC between 0.71 and 0.83. Moreover, we observe in Fig. 1 that the ΔE probability distributions of pathogenic and benign variants are well separated for the four tRNAs.

**Table 1.** Sensitivity, specificity, balanced accuracy, and area under the receiver operating characteristic curve of pycofitness in predicting the deleteriousness of variants with clinical annotations in non-coding RNAs.

Type	Sensitivity	Specificity	BACC	AUC-ROC	No. of variants
MT-TF	0.80	0.79	0.79	0.77	29
MT-TV	0.60	0.67	0.63	0.71	14
MT-TL1	0.69	0.80	0.74	0.83	26
MT-TI	0.71	0.66	0.69	0.70	16

We showcase two other applications of pycofitness in Supplementary Section S2. In the first, we used pycofitness to predict the impact of mutations on the fitness of four enzymes on which high-throughput fitness experiments have been performed (Weile *et al.* 2017), and compared its performance with that of six fitness predictors. We found that pycofitness achieves on average the same accuracy as the other unsupervised methods tested and, unsurprisingly, performs equally or slightly worse than the supervised methods, which are based on complex machine learning models and trained on huge amounts of variant information.

In the last application, we used pycofitness to predict the change in folding free energy (ΔΔG) upon all possible single-site mutations inserted in the $\beta_{1}$ domain of streptococcal protein G, and compared them with ΔΔGs measured by high-throughput experiments (Nisthal *et al.* 2019). We found that pycofitness scores correlate less well with experimental ΔΔGs than ΔΔGs obtained with the best supervised structure-based predictors. However, we showed how we can integrate the pycofitness scores into these other predictors to boost the overall performances.

In summary, we showed that pycofitness performs very well in protein and RNA fitness and deleteriousness predictions. For protein ΔΔG predictions, it performs less well than supervised structure-based methods dedicated to ΔΔG predictions. This is not surprising as pycofitness is based on evolutionary information and thus predicts fitness rather than stability, which are only partially correlated. For example, functional residues are highly conserved but not at all optimized for stability, usually being stability weaknesses of the protein structure (Ferreiro *et al.* 2018, Hou *et al.* 2021). However, pycofitness scores and predicted ΔΔGs are complementary and their combination yield increased predictions accuracy.

Detailed results of these analyses are available in our GitHub repository at https://github.com/KIT-MBS/pycofitness.

## 5 Conclusion

In this applications note, we have introduced pycofitness, a Python package for in-silico mutagenesis of RNA and protein sequences which uses an MSA of homologous sequences as input, and made it available to the scientific community. Its user-friendly installation, ease of use, and good performances make it very valuable, by itself or in combination with other tools, in a wide range of applications ranging from the interpretation of genetic variants to the rational modification of proteins and RNAs. We would like to emphasize that pycofitness is unsupervised and thus does not suffer from bias issues.

## Supplementary Material

btae074_Supplementary_Data

## Data Availability

The data underlying this article are available in the article, in its online supplementary material and in the repository https://github.com/KIT-MBS/pycofitness.
