# Supplementary material for: pycofitness—Evaluating the fitness landscape of RNA and protein sequences
Source: Bioinformatics. 2024 Feb 9;40(2):btae074. doi: 10.1093/bioinformatics/btae074 (PMC10881095; doi:10.1093/bioinformatics/btae074)
Supplement: btae074_Supplementary_Data [file btae074_supplementary_data.pdf]

Supplementary Information

pycofitness—Evaluating fitness landscapes of RNA and protein sequences

F. Pucci[†], M. B. Zerihun[†], M. Rooman, A. Schug

[†]Equally contributing

Table of content

- Section 1. Implementation of pycofitness [pages 2-4]
- Section 2. Examples of pycofitness applications [pages 4-7]
- Section 3. Comparison between DCA method inference approaches [page 7]

1 Implementation of pycofitness

Installation

pycofitness is freely available from the repository github.com/KIT-MBS/pycofitness. The software is also available from the Python package indexing (PyPI) and can be installed, for example, using *pip*:

```
pip install pycofitness
```

Input MSA

The input data for pycofitness is an MSA file in fasta format. We chose not to include alignment and curation tools in pycofitness, because in this way the users can employ their favorite alignment methods, e.g., [1, 2] and, in addition, curate and correct the input MSA by hand [3]. Note that one mandatory modification must be done to the input MSA:

- The target sequence to be mutated has to be the first entry of the MSA.

Method pipeline

The pipeline of pycofitness is illustrated in Fig. S1. It is based on two computational steps. The first is the inference of the DCA parameters (local field strength $h_i(a_i)$ and coupling strengths $J_{ij}(a_i, a_j)$) introduced in eq. (3) of the main text. To perform this step, we used the pseudo-likelihood maximization algorithm that has been introduced in [4] and has proven to be accurate to infer model parameters when applied to proteins [5, 6, 7] and RNAs [8, 9]. In pycofitness we used the implementation we developed in pyDCA [10]. The presentation of the pseudolikelihood maximization DCA is outside the scope of this manuscript and we refer the reader to [4] for the explanation of the algorithm and to [10] for technical information about our implementation.

The second step of the pycofitness pipeline starts once the parameters of the model, $h_i(a_i)$ and $J_{ij}(a_i, a_j)$, are inferred. It consists in performing in-silico mutagenesis, where the effect on the evolutionary energy of all single site mutations inserted at each position is computed using eq. (4) of the main text.

Command line interface

Once pycofitness is installed, it can be used from its command line interface, which allows the user to control the input parameters and to receive log messages to track the different steps of the computation. Below we list the commands to compute the evolutionary energy and all mandatory and optional input parameters:

- *Mutagenesis command.* In order to compute the effect of all single-site mutations on a given protein sequence, the command to use is:

pycofitness <biomolecule> <msa_file> [options].

where <biomolecule> is either "protein" or "rna" and <msa_file> the input MSA file. The optional commands are:

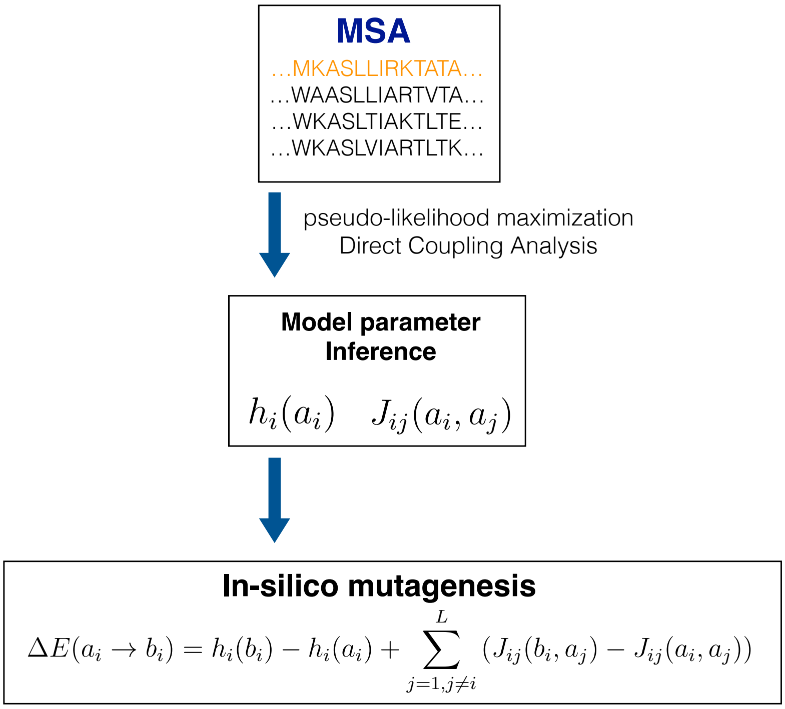

Figure 1: Schematic representation of the pipeline used by pycofitness to perform computational mutagenesis starting from the MSA of protein or RNA sequences homologous to the target.

- `--seqid`: sequence identity threshold for the clustering of the sequences in the input MSA. After clustering, the sequences are weighted according to the number of sequences in the corresponding cluster.
- `--lambda_h`: value of the penalizing constant for the local field strengths h for L2 regularization.
- `--lambda_J`: value of the penalizing constant for the coupling strengths J for L2 regularization.
- `--max_iterations`: maximal number of iterations for the gradient descent in the negative pseudolikelihood minimization algorithm.
- `--num_threads`: number of threads used in the pseudolikelihood minimization computation.
- `--verbose` : enables log messages to be displayed on the terminal.
- `--output_dir`: path to the directory where the output results are written. If the directory does not exist, it is created. If this path is not provided, an output directory is created with the base name of the MSA file, with *output_* prefix added to the MSA file base name.

pycofitness as a Python library

The software can be imported into user-written Python source codes as follows:

```python
from pycofitness.mutation.mutation import PointMutation

point_mutation = PointMutation(msa_file, biomolecule)
delta_phi = point_mutation.delphi_epistatic()
```

Values for the optional parameters can also be passed to the *PointMutation* constructor, for example:

```python
point_mutation = PointMutation(msa_file, biomolecule,
    max_iterations = 1000,
    num_threads = 4,
    seqid = 0.9,
    lambda_J = 10.0,
    lambda_h = 5.0,
    verbose = True
)
```

More detailed information on how to use the software can be found in the GitHub repository https://github.com/KIT-MBS/pycofitness.

2 Examples of pycofitness applications

We present in the main text an application of pycofitness for the deleteriousness prediction of mutations inserted in four mitochondrially encoded transfer RNAs. Here we present two additional mutagenesis applications of pycofitness, focused on protein fitness and protein thermodynamic stability. The aim of this section is to show the area of applicability of the pycofitness model and its good prediction performances compared to more complex models that use, for example, supervised deep learning algorithms and large amounts of training data. We would like to emphasize that pycofitness is unsupervised and thus does not suffer from bias issues.

Predicting fitness landscape

We used pycofitness to predict the impact of all possible single-site mutations in four proteins: SUMO E2-conjugating enzyme (UBE2I), small ubiquitin-related modifier 1 (SUMO1), thiamin pyrophosphokinase 1 (TPK1), and calmodulin 1 (CALM1). We started by performing a homology search of the target sequence in UniRef90 [11] and aligned the collected sequences using jackhmmer [12] with standard parameters, i.e. 5 homologous sequence search iterations. The MSAs so obtained were curated by removing all columns corresponding to gaps in the target sequence and then used as input of pycofitness which was run with default parameters.

We compared the pycofitness predictions with the experimental values obtained from deep mutagenesis scanning experiments [13], as well with six other fitness prediction methods, i.e. GEMME [14], EVCouplings [7], PROVEAN [15], AlphaMissense [16], DEOGEN2 [17] and ESM1b [18]. GEMME results have been computed using its webserver; PROVEAN, AlphaMissense and DEOGEN values have been downloaded directly from their respective repository; EVCouplings and ESM1b have been installed and run locally. Note that pycofitness, GEMME, PROVEAN, EVCouplings and ESM1b are unsupervised and do not use any variant information in their model construction. In contrast,

AlphaMissense and DEOGEN2 are based on complex machine learning models and use variant information for their training. The comparison between the performances of these two classes of methods must therefore be considered with caution.

	CALM1	SUMO1	TPK1	UBE2I	Average
pycofitness	0.25	0.45	0.24	0.50	0.360
EVCouplings	0.24	0.48	0.24	0.49	0.363
GEMME	0.23	0.52	0.24	0.48	0.368
PROVEAN	0.13	0.47	0.23	0.42	0.313
ESM1b	0.25	0.43	0.29	0.40	0.343
DEOGEN2	-	0.43	0.22	0.44	0.363*
AlphaMissense	0.19	0.55	0.31	0.55	0.400
# entries	1813	1700	3181	2563	

Table S1: Spearman correlation coefficient between the experimental fitness scores measured in [19, 13] for four proteins (SUMO1, CALM1, TPK1 and UBE2I) and the scores predicted by seven computational methods, among which five unsupervised (pycofitness, EVCouplings [7], GEMME [14], and PROVEAN [15], ESM1b [18]), and two supervised (DEOGEN2 [17], AlphaMissense [16]). *Average computed on only three values instead of four; indeed, DEOGEN2 values are unavailable for CALM1.

We report in Table S1 the Spearman correlation coefficient between the experimental fitness values of all single-site variants of the above-mentioned proteins [13], normalized as in [19], and the predicted fitness scores of pycofitness and the six other tested methods. It is interesting to observe that the scores of the tested methods differ according to the proteins, but reach a similar accuracy on the average. Indeed, the average Spearman correlation coefficient over the four proteins, consisting of a total of almost 10k mutations, is about 0.35. Although these values may seem low, we would like to emphasize that most of the methods (pycofitness, EVCouplings, GEMME, PROVEAN, ESM1b) do not use any variant data to train their model or fit parameters. Combining their predictions with other evolutionary or structural features has already been shown to substantially improve prediction accuracy [19, 20]. Note that EVCouplings and pycofitness almost perfectly agree. This is expected since they are basically two different implementations of the same pseudo-likelihood maximization DCA algorithm.

The supervised prediction methods DEOGEN2 and AlphaMissense, although using complex machine learning models trained on variant fitness information, achieve equal or slightly higher performance with average Spearman correlation coefficients of 0.36 and 0.40, respectively.

Finally, we want to underline that the performance of all MSA-based methods, including EVCouplings, pycofitness, DEOGEN2, GEMME and PROVEAN, crucially depend on the input MSA and its curation. In our benchmark, each method constructs its own input MSA from the target sequence in a slightly different way, which has undoubtedly an impact on the benchmark results. Similar issues have been discussed in [21].

Predicting protein thermodynamic stability

Rational modulation of thermodynamic stability is one of the key objectives of protein design, since low stability is often a major bottleneck in biotechnological processes. For

	PoPMuSiC	MAESTRO	DDGun3D	PremPS	pycofitness
r	0.56	0.58	0.59	0.57	0.31
σ	0.96	0.91	0.99	0.95	-
$\sigma_{+\text{pycofitness}}$	0.84	0.79	0.81	0.86	-

Table S2: Pearson correlation coefficient r and root mean square deviation σ (in kcal/mol) between predicted and experimental $\Delta\Delta G$ values of all single-site mutations inserted in the $\beta 1$ extracellular domain of streptococcal protein G; $\sigma_{+\text{pycofitness}}$ means the root mean square deviation of the model defined by eq. (1).

this reason, a wide series of computational predictors have been developed in the last decades to study the impact of mutations on the folding free energy ΔG [22].

We assessed here the performance of pycofitness in predicting the change in folding free energy upon mutations, noted $\Delta\Delta G$. We used as benchmark the experimental $\Delta\Delta G$ values for all single site mutations inserted in the $\beta 1$ extracellular domain of streptococcal protein G (PDB code 1PGA), which have been measured in [23]. We call S830 this set of 830 mutations. The pycofitness score computations were performed as described in the previous subsection: the input MSA was prepared by searching UniRef90 [11] for sequences homologous to the target, which were aligned using jackhmmer [12]; this MSA was used as input of the pycofitness mutagenesis command.

For comparison, we also assessed four predictors of protein stability changes upon mutation on the same test set: PoPMuSiC [24], MAESTRO [25], DDGun3D [26] and PremPS[27], selected as those performing the best on S830 [22]; we did not consider the DeepDDG method as it is extremely slow and cannot be applied on a large scale. We reported in Table S2 the Pearson correlation coefficients r and the root mean square deviation σ between the experimental and predicted $\Delta\Delta G$ values for pycofitness and the four tested stability predictors.

The results in Table S2 show that pycofitness underperforms on S830 mutations compared to the other tested methods. The fact that pycofitness is not as good as predictors dedicated to protein stability is not surprising. Indeed, pycofitness is based on evolution and thus predicts fitness rather than stability. These two quantities are far from perfectly correlated. For example, functional residues are highly conserved but not at all optimized for stability, usually being stability weaknesses of the protein structure [28, 29].

Moreover, the stability predictors tested are based on complex supervised machine learning approaches, use a series of different sequence and structural features, and require the 3D protein structure of the wild-type protein as input. In contrast, pycofitness is unsupervised and based solely on protein sequences. Considering this, pycofitness can be viewed as providing a good estimation of $\Delta\Delta G$.

To show how pycofitness can be used to improve stability predictions, we constructed four simple models that linearly combine the output of each of the four tested $\Delta\Delta G$ predictors with the pycofitness score as:

$$\Delta\Delta G_{pred} = \alpha\, X + \beta\, \text{pycofitness} \tag{1}$$

where X is the $\Delta\Delta G$ predicted by the method considered, and α and β two parameters optimized in cross validation at residue level on S830. We see in Table S2 that the models that include the pycofitness score have a root mean square deviation $\sigma_{+\text{pycofitness}}$ about 15% lower than the original predictors.

Finally note that, as already discussed in previous investigations [30, 31, 32], stability predictors are often biased towards destabilizing mutations because their training sets are usually strongly enriched in such mutations. As a result, they are more accurate on direct mutations ($A \to B$), which are often stabilizing, than on inverse mutations ($B \to A$), which are often destabilizing, although in principle we should have the equality:

$$\Delta\Delta G(A \to B) = -\Delta\Delta G(B \to A). \quad (2)$$

Since pycofitness satisfies this equation by construction, as visible in eq. (3) of the main text, it is perfectly antisymmetric and has thus the advantage of not suffering from biases towards destabilizing mutations.

3 Comparison between DCA inference approaches

We compared the performances of the *in silico* mutagenesis computations using different DCA approaches to infer the parameters h_i and J_{ij} of the coevolutionary model. More specifically, we compared the experimentally measured impact of all single-site mutations in UBE2I on its fitness [13] with the predictions obtained with six DCA approaches: an independent-site model, mean-field DCA (mfDCA) [33, 10], pycofitness and EVCouplings [34] that both use a plmDCA approach [4], Boltzmann machine DCA [35], and ArDCA [36].

Method	Correlation	time (s)
Independent-site	0.33	~ 10
mfDCA	0.39	$\sim 0.5 \text{ x } 10^3$
plmDCA (pycofitness)	0.50	$\sim 10^3$
plmDCA (EVCouplings)	0.49	$\sim 1.7 \text{ x } 10^3$
ArDCA	0.49	$\sim 1.6 \text{ x } 10^3$
bmDCA	0.45	$\sim 10^5$

Table S3: Spearman correlation coefficient between different DCA-based mutagenesis predictions and the experimental fitness values obtained from deep mutagenesis scanning experiments [13] for 2563 single-site mutations in UBE2I. Time is the time to do the inference of DCA-parameters, which is the bottleneck of the computation.

The results reported in Table S3 show that plmDCA and ArDCA reach the best performance, and especially its pycofitness implementation. bmDCA and mfDCA have a lower accuracy of about 10% and 20%, respectively; the latter is, however, the fastest approach. As expected, the independent-site model does not perform very well in comparison with the other models that are more complex and take residue coevolutions into account.

References

[1] Kazutaka Katoh and Daron M Standley. MAFFT multiple sequence alignment software version 7: improvements in performance and usability. *Molecular biology and evolution*, 30(4):772–780, 2013.

[2] Mark A Larkin, Gordon Blackshields, Nigel P Brown, R Chenna, Paul A McGettigan, Hamish McWilliam, Franck Valentin, Iain M Wallace, Andreas Wilm, Rodrigo Lopez, et al. Clustal W and Clustal X version 2.0. *bioinformatics*, 23(21):2947–2948, 2007.

[3] Charlotte Tumescheit, Andrew E Firth, and Katherine Brown. CIAlign: A highly customisable command line tool to clean, interpret and visualise multiple sequence alignments. *PeerJ*, 10:e12983, 2022.

[4] Magnus Ekeberg, Cecilia Lövkvist, Yueheng Lan, Martin Weigt, and Erik Aurell. Improved contact prediction in proteins: using pseudolikelihoods to infer Potts models. *Phys. Rev. E - Stat. Nonlin. Soft Mat. Phys.*, 87(1):1–16, 2013.

[5] Magnus Ekeberg, Tuomo Hartonen, and Erik Aurell. Fast pseudolikelihood maximization for direct-coupling analysis of protein structure from many homologous amino-acid sequences. *Journal of Computational Physics*, 276:341–356, 2014.

[6] Christoph Feinauer, Marcin J Skwark, Andrea Pagnani, and Erik Aurell. Improving contact prediction along three dimensions. *PLoS Comput. Biol.*, 10(10):e1003847, 2014.

[7] Thomas A Hopf, Anna G Green, Benjamin Schubert, Sophia Mersmann, Charlotta PI Schärfe, John B Ingraham, Agnes Toth-Petroczy, Kelly Brock, Adam J Riesselman, Perry Palmedo, et al. The EVcouplings Python framework for coevolutionary sequence analysis. *Bioinformatics*, 35(9):1582–1584, 2019.

[8] Fabrizio Pucci, Mehari B Zerihun, Emanuel K Peter, and Alexander Schug. Evaluating DCA-based method performances for RNA contact prediction by a well-curated data set. *RNA*, 26(7):794–802, 2020.

[9] Eleonora De Leonardis, Benjamin Lutz, Sebastian Ratz, Simona Cocco, Rémi Monasson, Alexander Schug, and Martin Weigt. Direct-coupling analysis of nucleotide coevolution facilitates RNA secondary and tertiary structure prediction. *Nucleic acids research*, 43(21):10444–10455, 2015.

[10] Mehari B Zerihun, Fabrizio Pucci, Emanuel K Peter, and Alexander Schug. pydca v1.0: a comprehensive software for direct coupling analysis of RNA and protein sequences. *Bioinformatics*, 36(7):2264–2265, 2020.

[11] Baris E Suzek, Yuqi Wang, Hongzhan Huang, Peter B McGarvey, Cathy H Wu, and UniProt Consortium. UniRef clusters: a comprehensive and scalable alternative for improving sequence similarity searches. *Bioinformatics*, 31(6):926–932, 2015.

[12] Robert D Finn, Jody Clements, and Sean R Eddy. HMMER web server: interactive sequence similarity searching. *Nucleic acids research*, 39(suppl_2):W29–W37, 2011.

[13] Jochen Weile, Song Sun, Atina G Cote, Jennifer Knapp, Marta Verby, Joseph C Mellor, Yingzhou Wu, Carles Pons, Cassandra Wong, Natascha van Lieshout, et al. A framework for exhaustively mapping functional missense variants. *Molecular systems biology*, 13(12):957, 2017.

[14] Elodie Laine, Yasaman Karami, and Alessandra Carbone. GEMME: A simple and fast global epistatic model predicting mutational effects. *Molecular biology and evolution*, 36(11):2604–2619, 2019.

[15] Yongwook Choi and Agnes P Chan. PROVEAN web server: A tool to predict the functional effect of amino acid substitutions and indels. *Bioinformatics*, 31(16):2745–2747, 2015.

[16] Jun Cheng, Guido Novati, Joshua Pan, Clare Bycroft, Akvilė Žemgulytė, Taylor Applebaum, Alexander Pritzel, Lai Hong Wong, Michal Zielinski, Tobias Sargeant, et al. Accurate proteome-wide missense variant effect prediction with AlphaMissense. *Science*, page eadg7492, 2023.

[17] Daniele Raimondi, Ibrahim Tanyalcin, Julien Ferté, Andrea Gazzo, Gabriele Orlando, Tom Lenaerts, Marianne Rooman, and Wim Vranken. DEOGEN2: prediction and interactive visualization of single amino acid variant deleteriousness in human proteins. *Nucleic acids research*, 45(W1):W201–W206, 2017.

[18] Nadav Brandes, Grant Goldman, Charlotte H Wang, Chun Jimmie Ye, and Vasilis Ntranos. Genome-wide prediction of disease variant effects with a deep protein language model. *Nature Genetics*, pages 1–11, 2023.

[19] Magnus Haraldson Høie, Matteo Cagiada, Anders Haagen Beck Frederiksen, Amelie Stein, and Kresten Lindorff-Larsen. Predicting and interpreting large-scale mutagenesis data using analyses of protein stability and conservation. *Cell Reports*, 38(2):110207, 2022.

[20] Matsvei Tsishyn, Gabriel Cia, Pauline Hermans, Jean Kwasigroch, Marianne Rooman, and Fabrizio Pucci. FiTMuSiC: Leveraging structural and (co) evolutionary data for protein fitness prediction. *bioRxiv*, pages 2023–08, 2023.

[21] Marina Abakarova, Céline Marquet, Michael Rera, Burkhard Rost, and Elodie Laine. Alignment-based protein mutational landscape prediction: doing more with less. *Genome Biology and Evolution*, 15(11):evad201, 2023.

[22] Fabrizio Pucci, Martin Schwersensky, and Marianne Rooman. Artificial intelligence challenges for predicting the impact of mutations on protein stability. *Current opinion in structural biology*, 72:161–168, 2022.

[23] Alex Nisthal, Connie Y Wang, Marie L Ary, and Stephen L Mayo. Protein stability engineering insights revealed by domain-wide comprehensive mutagenesis. *Proceedings of the National Academy of Sciences*, 116(33):16367–16377, 2019.

[24] Yves Dehouck, Jean Marc Kwasigroch, Dimitri Gilis, and Marianne Rooman. PoPMuSiC 2.1: a web server for the estimation of protein stability changes upon mutation and sequence optimality. *BMC bioinformatics*, 12(1):1–12, 2011.

[25] Josef Laimer, Heidi Hofer, Marko Fritz, Stefan Wegenkittl, and Peter Lackner. MAESTRO - multi agent stability prediction upon point mutations. *BMC bioinformatics*, 16(1):1–13, 2015.

[26] Ludovica Montanucci, Emidio Capriotti, Giovanni Birolo, Silvia Benevenuta, Corrado Pancotti, Dennis Lal, and Piero Fariselli. DDGun: an untrained predictor of protein stability changes upon amino acid variants. *Nucleic Acids Research*, 50(W1):W222–W227, 2022.

[27] Yuting Chen, Haoyu Lu, Ning Zhang, Zefeng Zhu, Shuqin Wang, and Minghui Li. PremPS: Predicting the impact of missense mutations on protein stability. *PLoS computational biology*, 16(12):e1008543, 2020.

[28] Diego U Ferreiro, Elizabeth A Komives, and Peter G Wolynes. Frustration, function and folding. *Current opinion in structural biology*, 48:68–73, 2018.

[29] Qingzhen Hou, Fabrizio Pucci, François Ancien, Jean Marc Kwasigroch, Raphaël Bourgeas, and Marianne Rooman. SWOTein: A structure-based approach to predict stability strengths and weaknesses of proteins. *Bioinformatics*, 37(14):1963–1971, 2021.

[30] Fabrizio Pucci, Katrien Bernaerts, Fabian Teheux, Dimitri Gilis, and Marianne Rooman. Symmetry principles in optimization problems: an application to protein stability prediction. *IFAC-PapersOnLine*, 48(1):458–463, 2015.

[31] Fabrizio Pucci, Katrien V Bernaerts, Jean Marc Kwasigroch, and Marianne Rooman. Quantification of biases in predictions of protein stability changes upon mutations. *Bioinformatics*, 34(21):3659–3665, 2018.

[32] Dinara R Usmanova, Natalya S Bogatyreva, Joan Ariño Bernad, Aleksandra A Eremina, Anastasiya A Gorshkova, German M Kanevskiy, Lyubov R Lonishin, Alexander V Meister, Alisa G Yakupova, Fyodor A Kondrashov, et al. Self-consistency test reveals systematic bias in programs for prediction change of stability upon mutation. *Bioinformatics*, 34(21):3653–3658, 2018.

[33] Faruck Morcos, Andrea Pagnani, Bryan Lunt, Arianna Bertolino, Debora S Marks, Chris Sander, Riccardo Zecchina, José N Onuchic, Terence Hwa, and Martin Weigt. Direct-coupling analysis of residue coevolution captures native contacts across many protein families. *Proc. Natl. Acad. Sci. U.S.A.*, 108(49):E1293–301, 2011.

[34] Thomas A Hopf, Anna G Green, Benjamin Schubert, Sophia Mersmann, Charlotta P I Schärfe, John B Ingraham, Agnes Toth-Petroczy, Kelly Brock, Adam J Riesselman, Perry Palmedo, Chan Kang, Robert Sheridan, Eli J Draizen, Christian Dallago, Chris Sander, and Debora S Marks. The EVcouplings Python framework for coevolutionary sequence analysis. *Bioinformatics*, 35(9):1582–1584, 10 2018.

[35] William P Russ, Matteo Figliuzzi, Christian Stocker, Pierre Barrat-Charlaix, Michael Socolich, Peter Kast, Donald Hilvert, Remi Monasson, Simona Cocco, Martin Weigt, et al. An evolution-based model for designing chorismate mutase enzymes. *Science*, 369(6502):440–445, 2020.

[36] Jeanne Trinquier, Guido Uguzzoni, Andrea Pagnani, Francesco Zamponi, and Martin Weigt. Efficient generative modeling of protein sequences using simple autoregressive models. *Nature communications*, 12(1):5800, 2021.
